# 1D Barcode Detection: Novel Benchmark Datasets and Comprehensive Comparison of Deep Convolutional Neural Network Approaches

**DOI:** 10.3390/s22228788

**Published:** 2022-11-14

**Authors:** Teerawat Kamnardsiri, Phasit Charoenkwan, Chommaphat Malang, Ratapol Wudhikarn

**Affiliations:** 1 Department of Digital Game, College of Arts, Media and Technology, Chiang Mai University, Chiang Mai 50200, Thailand; 2 Department of Modern Management and Information Technology, College of Arts, Media and Technology, Chiang Mai University, Chiang Mai 50200, Thailand; 3 A Research Group of Modern Management and Information Technology, College of Arts, Media and Technology, Chiang Mai University, Chiang Mai 50200, Thailand; 4 Department of Digital Industry Integration, College of Arts, Media and Technology, Chiang Mai University, Chiang Mai 50200, Thailand; 5 Department of Knowledge and Innovation Management, College of Arts, Media and Technology, Chiang Mai University, Chiang Mai 50200, Thailand

**Keywords:** barcode dataset, deep learning, convolutional neural network, barcode recognition, barcode detection, benchmarking

## Abstract

Recent advancement in Deep Learning-based Convolutional Neural Networks (D-CNNs) has led research to improve the efficiency and performance of barcode recognition in Supply Chain Management (SCM). D-CNNs required real-world images embedded with ground truth data, which is often not readily available in the case of SCM barcode recognition. This study introduces two invented barcode datasets: InventBar and ParcelBar. The datasets contain labeled barcode images with 527 consumer goods and 844 post boxes in the indoor environment. To explore the influential capability of the datasets that affect recognition process, five existing D-CNN algorithms were applied and compared over a set of recently available barcode datasets. To confirm the model’s performance and accuracy, runtime and Mean Average Precision (mAP) were examined based on different IoU thresholds and image transformation settings. The results show that YOLO v5 works best for the ParcelBar in terms of speed and accuracy. The situation is different for the InventBar since Faster R-CNN could allow the model to learn faster with a small drop in accuracy. It is proven that the proposed datasets can be practically utilized for the mainstream D-CNN frameworks. Both are available for developing barcode recognition models and positively affect comparative studies.

## 1. Introduction

In recent years, deep learning (DL) has been widely accepted and commonly applied in a variety of study fields more than other machine learning (ML) algorithms [1]. DL could provide outstanding performance in terms of quality, speed, precision, or accuracy across various applications and research domains. Based on its distinctive advantages and its practical uses in both real-life and experimental situations, DL has overcome other past well-known techniques. Thus, it has been highly adopted in several domains, such as communication systems [2], manufacturing and production system [3], finance [4], tourism [5], medical processing [6], computer games [7], bioinformatics [8], robotics [9], and so on. Similar to other research domains, supply chain management (SCM) could substantially benefit from adopting DL methods in a broad range of SCM parts and activities. Especially a barcode recognition task, which is identified as a backbone of SCM, could achieve its goals efficiently and effectively when applying the DL method. DL could improve both qualities of barcode images with better clearness, as well as fineness [10,11,12], and barcode analysis performance with greater accuracy and real-time performance [13,14,15].

Regarding the substantial benefits of DL, it has become widespread in the barcode recognition task in recent years. From past related studies, DL approaches applied to barcode analysis can be categorized into two major categories: the multi-layer perceptron (MLP) and convolutional neural networks (CNNs). From these two techniques, CNNs-based DL, also known as deep CNNs or D-CNNs, is more utilized than the MLP algorithm [1]. D-CNNs have outperformed MLP in several dimensions. One of its distinctive and superior capabilities over MLP is the improvement of information loss originating from converting two-dimension images to one-dimension signals [16]. Therefore, regarding the specific advantage of D-CNNs, this technique has been incorporated into various barcode recognition tasks, which can be categorized into two primary operations, including detecting and decoding processes. In the recent decade, several studies applied D-CNNs with barcode recognition tasks. Nevertheless, all the past attempts still involved two major limitations.

The first issue concerns the limited sources of public and realistic barcode datasets. Generally, in DL model development studies, an efficient dataset is crucial and highly required. Undeniably, the data used for training the model has substantial effects on the robustness of the developed DL method [17]. Similar to DL models in other fields, the development of D-CNN-based barcode recognition model requires a reliable, high-quality, and realistic dataset. Moreover, as our recent study [18] pointed out, the DL-based barcode recognition methods mainly rely on a large and high-quality dataset with ground truth data. Unfortunately, these barcode datasets are mostly not readily and publicly available for model training and testing, especially for free use. The public barcode datasets are currently faced with data annotation problems and are labor-intensive. Most of them are not ready to be used due to a lack of annotated data and require user manual labeling. Some public barcode datasets do not combine harsh conditions in real-world environments, causing biases in model training and barcode decoding. Although the existing private resource datasets are often generated to resolve all the above issues, accessing is not permitted. From the above limitations, they restrict scholars, as well as practitioners from accessing various high-quality and realistic datasets. These circumstances could consecutively obstruct them from sufficient training and testing and creating efficient DL models.

The latter limitation involves the limited adoption of D-CNN methods, which still could not cover a wide range of well-known approaches and their recent frameworks. Therefore, the limited realization of comprehensive D-CNN methods’ performances significantly obstructs the ability of scholars and practitioners to perceive the current optimum methods for the barcode recognition task. To the best of our knowledge [18], some well-known and efficient D-CNN frameworks, such as EfficientDet and RetinaNet, have not been utilized with the barcode recognition task. However, they were widely and mostly applied with image recognition tasks in other domains such as medicine, transportation, agriculture, etc. Furthermore, the adoption of recent and efficient frameworks of D-CNN methods is still neglected in past studies. For example, one of the most famous object recognition methods [19], You-Only-Look=Once (YOLO), has been widely applied in most D-CNN-based barcode recognition studies. Nevertheless, until now, the last version of YOLO, which was deployed to barcode recognition studies, was YOLO version 4 [14] despite the current YOLO version 6. However, YOLOv5 has been claimed as a game changer for several research domains and industries among the YOLO family [20]. It could bring several advantages and significantly better performance over the past versions, such as more accuracy [21], smaller size [20], and faster training [22]. Regarding the superior performance of the recent version and other underexplored D-CNN methods and, significantly, none of their application in the barcode recognition task, this important and critically limits the recognition and future improvement of capabilities of D-CNN-based barcode recognition.

To improve the major limitations of D-CNN-based barcode recognition mentioned above, in this study, we propose two novel barcode datasets named “InventBar” and “ParcelBar” for developing and investigating a robust DL-based barcode recognition model. The first dataset, InventBar, comprises 527 images captured from daily life consumer goods from supermarkets, and the second dataset, ParcelBar, consists of 844 images of parcels shot from post offices. As the traditional 1D barcodes are more commonly used and have long-range impacts in the SCM domain, the proposed datasets only emphasize the 1D barcodes. The datasets differ from the previous public barcode datasets, which are the real-life captured barcode images in the SCM domain. InventBar and ParcelBar contain a sufficient number of barcodes with different sizes of barcode regions and are provided with data annotations. Inspired by the assumption that real-world barcode images are not often of a high quality, our proposed datasets were created by involving five distinct quality distortions, i.e., light conditions, complex backgrounds, rotations, different sizes of bounding boxes, and blurry areas. These proposed datasets are publicly available and also made free of charge. The datasets containing the original barcode images and the respective annotations are available at https://cmu.to/BenchmarkBarcodeDatasets (created on 13 October 2022).

To perceive and compare the performance of the well-known state-of-the-art D-CNN architectures, secondly, we benchmark underexplored DL techniques for barcode recognition (i.e., YOLO v5 [23], YOLO x [24], EfficientDet [25], and RetinaNet [26]) with other previously and widely applied D-CNN methods (i.e., Faster Region Convolutional Neural Network or Faster R-CNN [27]). In this aspect, our work contributes to developing an alternative solution for barcode recognition. We examine the hypothesis that D-CNN-based barcode recognition algorithms can be optimized both in speed and accuracy for SCM applications, especially when using a set of well-defined barcode objects. Regarding our proposed improvements, the contributions of this study can be listed as follows.

1. Benchmarking recent state-of-the-art and underexplored D-CNN frameworks with other prior well-known solutions by utilizing the novel barcode datasets: InventBar and ParcelBar and other former public and realistic datasets.
2. Analyzing some significant characteristics of the recent publicly available barcode datasets corresponding with the application effects of the well-known D-CNNs on 1D barcode detection.
3. Collecting and maintaining the recent barcode datasets with well-completed annotations and partitioning them into a series of training, validation, and test sets; those are ready for use.
4. Evaluating both the performance and efficiency of all implemented D-CNN solutions.

The remaining parts of this study are organized as follows: Section 2 provides past studies on barcode datasets and applications of D-CNNs in barcode recognition. Section 3 describes the materials and methods adopted in this study, followed by the results discussed in Section 4. Finally, Section 5 concludes the research findings, limitations, and possible future works.

## 2. Related Works

### 2.1. Previous Barcode Datasets

Over seven decades ago, different barcode datasets were invented and adopted broadly in academic and commercial domains. Massive barcode data and the quality of barcode images have made new barcode recognition methods based on D-CNNs increasingly dominant. Barcode data played a key role in building an intelligent approach for barcode localization and decoding, while its quality is necessary for the D-CNNs to operate efficiently. It is undeniable that the more and better the barcode data we provide to the D-CNNs model, the faster the model can learn and improve.

In the field of SCM, it is common knowledge that the existence of Computer Vision (CV) methods, i.e., the DL allows substantial improvement and significantly enhances both ability and performance of barcode recognition and analysis. Several previous pieces of research have thoroughly examined and studied barcode recognition using D-CNN-based tools and techniques. Some studies also proposed barcode datasets that can be reused for developing barcode detection and analysis models [28,29,30]. Until now, two common classes of barcode datasets have been developed; those are public and private datasets. Public barcode datasets are datasets containing either synthetic barcode images or real-world captured barcode images. They were previously collected by research scholars or practitioners and made available for public use [31]. The private barcode datasets, on the contrary, are primary source barcode databases with restricted access. Apart from the above two classes, there is also the synthetic barcode or the computer-generated dataset. This class of barcode dataset requires less effort to obtain labeled barcode images, which also benefits the model development.

Current barcode recognition studies require a sufficient number of high-quality datasets for model training and benchmarking. However, most existing ones are not given instant access; they are private or unsearchable [32,33,34,35,36,37,38]. The public or online datasets can be easily accessed and freely utilized among different sources of barcode datasets. As declared in our previous study [18], the public datasets are denoted as the most often used ones. They receive more remarkable attention from scientific research than private barcode datasets. Despite the high accessibility, only a few public barcode datasets are currently available. The statistical evidence from barcode analysis research in 2017–2021 shows the three most frequently utilized barcode datasets, i.e., the well-known Art-Lab, Art-Lab Rotated, and WWU Muenster (accounting for more than 64%). This result could emphasize the lack of public barcodes and highlight the necessity of new public barcode datasets for SCM and related research areas.

To gain a broader perspective of the currently available barcode datasets, in this section, we give a brief overview of the existing public barcode datasets that play an important role in this research area. As illustrated in Table 1, there are nine publicly available barcode datasets, including the Arte-Lab Medium Barcode Dataset (Set1 and Set2) [39], Arte-Lab Rotated Barcode Dataset [40], WWU Muenster Dataset [41], 1D Barcode Extended Dataset [42], Dubska’ M Dataset [43], Sörös G, and Flörkemeier’s Dataset [29], Bodnár-Synthetic, and Bodnár-Huawei Dataset [30]. Detailed information about each dataset is also presented, for instance, the size of the dataset, the number of barcode images contained in each image, the pixel resolution of the barcode images, and different features of the barcode images.

The same group of researchers invented the first four datasets presented in Table 1. They are all maintained by the Applied Recognition Technology Laboratory, Department of Theoretical and Applied Science, University of Insubria [44]. The Arte-Lab Medium Barcode Datasets [39] can be separated into two sets. Both contain an equal number of barcode images captured by a Nokia 5800 mobile phone. Barcode images in the Arte-Lab Medium Barcode (Set 1) are taken from devices with autofocus, whereas Set 2 were collected using devices without autofocus. Each image contains at most one non-blurred EAN barcode. However, barcodes that appeared in Set 1 are rotated by, at most, ±30° from the vertical, enabling this dataset to not be suitable for evaluating the performance of angle invariant algorithms. Due to the lack of barcode resources and to serve barcode orientation detection, Zamberletti et al. [42] extended the original Arte-Lab dataset to include a few more barcode images in different rotation angles. The dataset is enclosed with binary images that allow defining the object region precisely. Another alternative dataset is the 1D Barcode Extended Dataset [42]. It was specifically proposed for evaluating the detection algorithms in the Hough transform space. The dataset comprises a subset of barcode images from Arte-Lab or some images captured from the same products presented in the Arte-Lab Rotated Dataset. Evidently, the barcode images and some characteristic appearances of all these datasets are identical, proving wholly inadequate. The datasets could not indeed feed the model with various barcode objects, which might be the biggest hindrance to the learning process for barcode recognition. Thus, it is required for the new barcode dataset that fully captures all new barcode images differently.

Apart from the limitation of public data, there are some challenges regarding the different sizes and dataset quality. The size of the barcode dataset is one of the biggest concerns for the efficient learning process. The D-CNNs always require a sufficient number of barcodes to reasonably approximate the unknown underlying mapping function from the input barcodes. However, as shown in Table 1, some searchable datasets are relatively small, comprising a hundred or less than hundreds of images (i.e., Bodnár-Huawei) that are further divided into a small training set and test set. It is worth reminding that insufficient training data will result in a poor approximation (either underfit or overfit the small training dataset). In contrast, too-small test data fundamentally allow an optimistic and high variance estimation [45,46]. To make the D-CNNs training possible, the majority of barcode recognition studies required a heavy data augmentations process [47,48], which can provide more representative training samples but consume more time and high computational complexity.

Another key success of barcode recognition also depends on the quality of barcode images and their influences on the model performance. In practical applications, the input images cannot always be assumed to be of high quality [49]. In computer vision applications, high-quality barcodes, e.g., clear backgrounds, simple patterns, and high-resolution images, do not confirm the recognition method’s performance. At the same time, barcode recognition in low-quality images is an important capability for D-CNNs. However, too complicated background, large image size, and a variety of barcode appearances might also bring D-CNNs learning and decoding tasks into a highly challenging procedure [11].

Regarding model training, there might appear to be a trade-off between the quality of datasets and model performance. As stated in [50], high image resolution for D-CNNs training directly affects the maximum possible batch size, causing a delay and high computational consumption. Moreover, a simple barcode image with a clear background or a large area of barcode objects might provide better accuracy but cause more overfitting [48]. Thanks to the research improvement in this area, as can be observed in Table 1, various existing barcode datasets are more focused on barcodes with specific features taken from real-life, most of which are imperfect or low-quality images. This way, the optimal selection of datasets containing different image features might significantly benefit the training and testing process rather than the high-quality images.

In addition, barcodes in some datasets, i.e., Dubská M., Bodnár-Synthetic, and Bodnár-Huawei, are not real-world samples, and the representation of barcodes does not even include real-life conditions. As seen in [51], their experiment was done over the Bodnár-Huawei dataset. The dataset contains computer-generated barcodes overlaid on the real background images instead of the fully captured real-world barcode images. This circumstance could also limit the capability of the D-CNN-based barcode recognition algorithms since the model has less opportunity to learn and improve from various distinct conditions of barcodes. When the datasets were applied to more specific analytical purposes, barcode recognition algorithms might fail to consider real-world characteristics and harsh conditions. Although many D-CNN methods have obtained state-of-the-art performance and can deal with the barcodes in different angles, shapes, and image quality, the methods might provide precise results at the experimental level but not the practical level.

It should also be carefully considered that the fully captured barcode image datasets are sometimes generated by adding adversarial objects, conflicted noises, or quality distortions from artifacts. These sources of noise are imperceptible to human observers, known as “worst noise” [49], which is the cause of deep learning networks misclassified [52]. In the same way, D-CNN may face difficulty predicting the correct class of barcode images under the worst noise. Encountering well-chosen noises while avoiding the worst noise is unlikely to be seen in the practical application and has become an interesting problem in the most recent research. We argue that the choice of barcode dataset containing well-captured images and some naturally quality distortions, e.g., illuminated, skewed, small, obscured, blurred, and rotated barcodes are preferable. It is a practical solution for developing the barcode recognition model that best fits the real-world situation.

Although real-life barcode images have gained more attention in the current public datasets, 66.67% of the freely available barcodes are labor-intensive because ground truth data is unavailable. The WWU Muenster dataset is one of the high-quality datasets since it was established under the actual situation and contains a sufficient number of train and test images. However, the dataset still required manual labeling by workers to complete the annotation task.

As a matter of fact, research in this area constantly needs more new images and a large enough barcode dataset that can efficiently enhance the model development process. A large margin of real-world barcode datasets should promote the accuracy and performance of D-CNN-based on barcode recognition. Considering the barcode images with real situations collected from the actual SCM environment, together with the well-chosen distortions, are the most necessary. With the support of our proposed datasets, we anticipated that the D-CNN-based barcode recognition technology could provide significant progress for detecting and decoding functions.

### 2.2. Deep Learning (DL) and Convolutional Neural Network (CNNs) for Barcode Recognition

Deep learning (DL) has come to be known as deep structured learning. The DL technique is considered a specific subfield of machine learning (ML) endowed with artificial neural networks (ANNs) to enable machines to make accurate decisions without relying on human supervision [53]. DL has attracted great attention in recent research, because it can efficiently resolve real-life problems and present great promise as a practical solution in several domains or situations. In computer vision domains, DL has been reported to outperform traditional approaches in object segmentation, image classification, and recognition [54]. Additionally, the advantage of DL could be extended to biological domain [55], computer games [56], communication systems [57], mobile traffic classification [58], and IoT-based UAV systems [59], as well as the named entity recognition [60].

Among various research fields, barcode recognition is one of the significant domains adopting DL and can receive better advantages than the traditional approaches. Several proposed and applied DL architectures can be classified into two primary techniques: multi-layer perceptron (MLP) and convolutional neural networks (CNNs), known as Deep CNNs or D-CNNs. However, from these two methods, D-CNNs are identified as more utilized DL algorithms [1] in the barcode analysis, since they can better resolve the information loss emerging from the conversion of two-dimensional images to one-dimensional vectors than the MLP architecture [16]. Moreover, D-CNNs also could better deal with other critical issues of barcode recognition and analysis, such as image blurring and image distortion [11,61]. Therefore, regarding the distinctive advantages of D-CNNs and the advancement of hardware, several studies have adopted this approach in recent years. Table 2 summarizes studies that apply D-CNN-based on barcode recognition methods in the barcode recognition field.

From Table 2, it can be seen that the main D-CNN methods that were employed over 2015–2021’s barcode studies include CNNs, SSD, R-CNN, Fast R-CNN, Faster R-CNN, DSC, and different versions of YOLO, ranging from version 2 (v2) to version 4 (v4). These DL methods can be classified into two major categories of object detectors; multiple-stage and single-stage detectors [76]. The multiple-stage method, mainly two-stage detectors, such as CNNs, R-CNN, Fast R-CNN, and Faster R-CNN, generates regions of interest before defining candidate bounding boxes. On the other hand, single-stage detectors, such as YOLO, and SSD, execute bounding-box regression and object classification simultaneously. Regarding their distinctiveness, typically, the multiple-stage detectors can reach higher localization and accuracy rates, while their speed is lower than the single-stage detectors.

From the different applications of D-CNN methods in the barcode recognition study presented in Table 2, CNN was the most frequently applied to this topic (10 out of 26 papers). Conversely, YOLO was denoted as the second most used technique (six papers). Nevertheless, as our past study [18] indicated, the analysis result shows a significant drop in D-CNNs utilization during 2020–2021 compared to the previous periods between 2015 and 2019. Especially considering the proportion of each popular method applied between the most recent year (2021) and whole years (2015–2021), YOLO was utilized more than 30%, while CNN adopted only 10%. The significant decline of CNN attention and application mainly comes from the fundamental issues of multiple-stage detectors, especially the more complex process and low-speed detection rate that do not meet both actual industrial requirements and real-life usages [77,78].

On the other hand, when focusing on 2021, YOLO architecture was the most applied method, taking more than 66% of articles related to the barcode recognition and analysis tasks. This declaration also emphasizes the single-stage detector in the barcode recognition task. Nevertheless, to the best of our knowledge, several approaches to single-stage detectors are recently, widely, and continuously adopted. Until now, some of the latest approaches, such as EfficientDet (popular in the biological domain), RetinaNet (widely used for detecting objects in aerial and satellite imagery), and the earliest version of the existing YOLO, are mostly claimed for better performance but have still never been explored in the barcode recognition research.

Therefore, regarding the limitation of applying modern and widely acknowledged approaches of D-CNNs, in this study, we adopt five representative object detection-based D-CNN methods, including the prior well-known and distinctive SCM solutions, i.e., Faster R-CNN [27] and a set of underexplored methods, which are EfficientDet [25], RetinaNet [26], YOLO v5 [23], and YOLO x [24], to comprehensively perceive and benchmark the effectiveness and efficiency of various D-CNN approaches.

## 3. Materials and Methods

### 3.1. Experimental Settings

This section introduces an outline process and methodologies used in this study. There are three key processes: data annotations, transfer learning, and model training and testing. A detailed explanation for each process will also be described. We applied five D-CNN-based methods and investigated key characteristics and quality of seven benchmark barcode datasets using different evaluation metrics. We used a Windows 10 OS laptop computer equipped with Intel(R) Core(TM) i5-8265U CPU@1.60 GHz, 2 GB NVIDIA graphic card, and 8 GB DDR4 RAM (ASUSTek Computer Inc., Taipei, Taiwan) for exploring, prototyping, and tuning hyper-parameter. The model training and testing were performed on the Kaggle web-based data-science environment (https://www.kaggle.com/) that offers a P100 GPU with 16 GB memory on Intel(R) Xeon(R) CPU@2.30 GHz model (accessed on 13 April 2022).

### 3.2. Dataset Description

As described in the previous section, few datasets deal with detecting barcodes in a specific SCM domain. Zamberletti et al. [39] presented the Medium Barcode 1D Collection, known as the Arte-Lab Barcode Dataset, which contains only book barcode images. In line with this, the Arte-Lab Rotated Barcode Dataset has been proposed as an extension. The new version of Arte-Lab contains rotated book barcodes from different angles and comprises a few barcodes from daily life products. Although the 1D Barcode Extended Dataset contains consumer-packaged goods barcodes [42], their provided barcode objects are not varied, most of which are images taken from a single consumer good with distinctive positions. Additionally, there is the dataset proposed for deblurring algorithms [29]. The dataset comprises blurry barcode images captured with intentions, thus far from everyday images. Some other existing datasets, such as Bodnár [30] and Dubská M. [43], encompassed the computer QR codes on both artifacts and real-world background images. Dissimilar to the WWU Muenster dataset [41], which is more probably to provide a high-feature representation of SCM objects with real scenarios. Obviously, most of the existing datasets show no sign of real-life SCM barcode objects captured from a variety of products. Moreover, none of the abovementioned datasets offer a comprehensive range of barcode tags on parcels from the express delivery service. These matters might limit building computational solutions for barcode analysis and recognition in the daily SCM environment.

To uncover the issues above, we present two new barcode datasets, the InventBar dataset, and the ParcelBar dataset. The main purpose of giving these two datasets is to provide a new set of barcode images with the presence of real or natural conditions that could also benefit the SCM and computer science communities. These two barcode recognition datasets specifically deal with SCM-related objects in the presence of indoor scenes. In the data collection process, all barcode images were collected manually using Samsung Galaxy S10 Plus with a 16 MP (f/2.2) ultrawide camera. We easily capture all barcode images within a short distance, ranging from an inch to a few feet. Barcode images with complex natural backgrounds, skews, blurry regions, and lighting conditions were grabbed, representing the most common real-world features. This operation would allow the model to deal with a higher challenge in barcode quality but prove the strengths of D-CNNs in 1D barcode recognition. We hoped that both InventBar and ParcelBar could serve as the basis for the D-CNN-based barcode detection and decoding approaches that can support further research on daily life barcodes in SCM.

### 3.3. Data Annotations

Inventing a new barcode dataset required the most expensive steps to manually label all collected barcode images by annotators [79]. The data-labeling process aims to provide a bounding box for the barcode in each photograph. Our InventBar and ParcelBar are one-class labeled datasets where all data corresponds to the axis points of the barcode region. The formerly proposed dataset, InventBar, is a collection of unique product identifiers ready to be sold in grocery stores. All barcode images are positives containing 1D barcodes with purely unique numbers. The latter dataset, ParcelBar, contains post-box tags collected from the indoor logistic warehouse. All datasets contain images captured from mobile cameras; thus, each image encloses either one or several barcode tags.

Before annotating the data, we performed a data cleaning process over raw datasets by removing duplicated images containing exactly the same instances captured at a similar angle. In our case, the duplicated barcode images are unintentionally taken from the burst mode. This preprocessing step resulted in 527 images of the InventBar dataset with relatively high quality (4032 × 3024 pixels), whereas ParcelBar involves 844 images in originally 1478 × 1108-pixel dimensions. There are 527 and 1088 barcode instances for InventBar and ParcelBar, respectively.

After that, we used the open-source software LebelImg V1.8.0 (https://sourceforge.net/projects/labelimg.mirror/files/v1.8.0/, accessed on 13 October 2022) to annotate all original barcode samples. The barcode instances are covered with the rectangular bounding box corresponding to four fundamental values, including x1, y1, x2, and y2, where x1 and y1 indicate the upper-left corner of the bounding box. It is noticeable that the data-labeling process significantly affects the level of detection accuracy. With a small mistake on the data label, the D-CNN models cannot effectively learn the ground truth, leading to fault detection. To ensure a high-quality annotation, two additional machine learning and deep learning practitioners participated in cross-checking and verifying the correctness of the barcode labels. In this regard, mislabeled barcode instances should also be reported and adjusted promptly.

We investigated the barcode tags based on the wrapped bounding box area for a detailed analysis of the significant features of barcode datasets. Based on the COCO 2017 dataset [80], barcodes in all images can be classified into small, medium, and large bounding box regions. As can be observed in Table 3, InventBar and ParcelBar show a greater proportion of large-sized barcode tags (accounted for 86.14% and 67.28%, respectively). In comparison, only 26.56% of the overall barcode instances are considered medium. It is also clear that both datasets show no sign of the small-sized barcodes.

**Figure 1:**
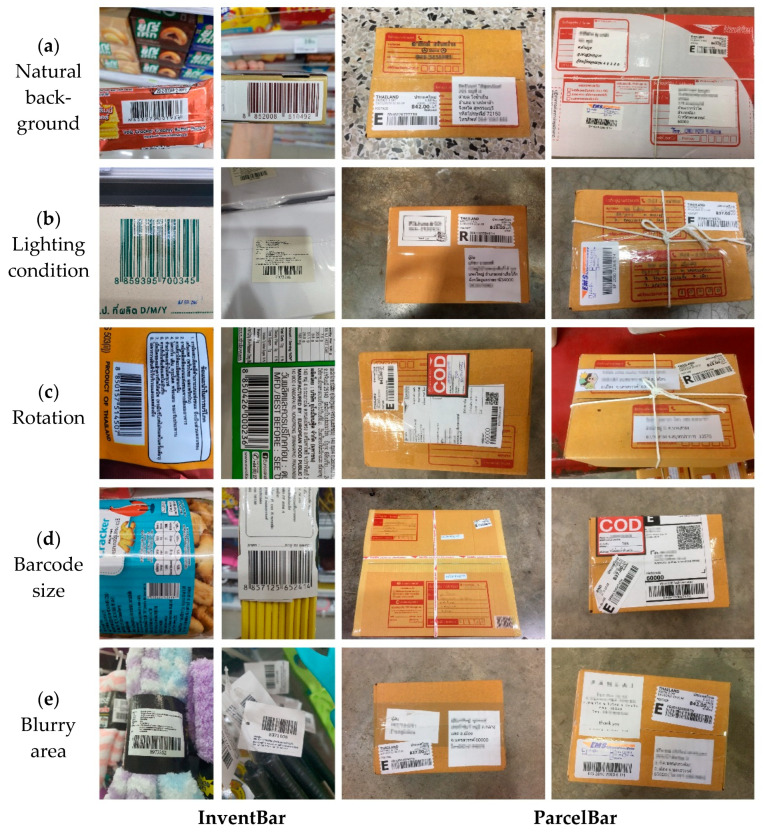
Example barcode images from the InventBar and ParcelBar datasets with distinctive natural characteristics: (**a**) natural background, (**b**) lighting conditions, (**c**) rotation, (**d**) barcode size, and (**e**) blurry area.

In accordance with the illustrations shown in Figure 1, our datasets not only present a barcode region in different scales but also involves diverse background texture from natural scenes or real-world SCM environments, such as the ground floor, products on the shelves, plain post boxes, and striped boxes with rope and messy characters. These key features make our proposed datasets complete and most suitable for training the barcode recognition algorithms.

### 3.4. Transfer Learning

After manually labeling the barcode datasets, transfer learning was utilized to fine-tune D-CNN-based barcode recognition models to realize accurate detection of barcode objects [77] and to accelerate the training time of all comparative models. It is a helpful technique that allows D-CNN-based methods to learn from a limited amount of data [81] but can still achieve a better result and with more computationally efficient [82]. By applying transfer learning in this study, the adopted D-CNN methods can perform a new task (detecting barcode objects) based on the knowledge from the previous well-trained models in different but related problems [83]. Accordingly, we used an IceVision pretrained framework over a large-scale object detection dataset, namely MS COCO 2017 (Microsoft Common Objects in COntext) [80], using different backbones shown in Table 4. The dataset comprises various image classes, such as persons, cars, and animals, with annotations for object attributes.

### 3.5. Model Training and Testing

**Figure 2:**
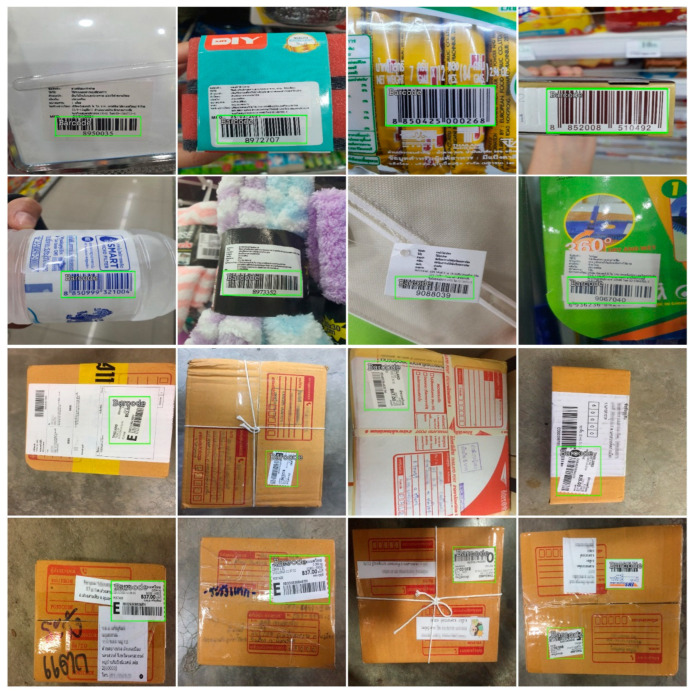
Image of the training data corresponds to the InventBar and ParcelBar and their respective annotations.

In the training process, we have trained and tested five D-CNN network models over a set of benchmarking datasets. This process also includes barcode data for InventBar and ParcelBar, as given in an example in Figure 2. The representative D-CNN methods can be classified into two groups. The mainstream group methods were previously applied in barcode recognition or one of the SCM solutions, including Faster R-CNN and the YOLO family. The methods in the second group are taken across the study domain, most of which are used in a field of biology, i.e., RetinaNet and EfficientDet. D-CNN-based barcode recognition models were trained and tested on different augmentation modes, including resizing, horizontal and vertical flip, shift scale rotation, RGB shift, and random brightness. All models either learned or verified on the well-defined transformation loop, ranging from 0 (without augmentation), 5, 10, and 20 (the highest augmentation value). For each comparable dataset, we randomly divided the total number of training samples into different batches but with the same size (eight samples/batch). The epoch numbers are set to 25, 50, and 100 to observe the data diversity and the iterative process’s impact, while the discriminator network’s learning rate is set to 0.001. We set up the rest of the required parameters in each algorithm to their default values of the networks. The trained models were further tuned for the highest precision and recall rate, which varies between 0 and 1, using the validation set. Table 5 summarizes the general information on all benchmarked datasets split into three subsets (i.e., training, validation, and testing sets with ratio 40:40:20) using random selection.

Theoretically, the number of samples and image resolution of different barcode datasets significantly affect model training. When the number of barcode images is too large with a high pixel degree, it could impair the performance of D-CNN-based barcode detection. It is worth noting that in common object detection algorithms, different images vary in lengths and widths. Moreover, the D-CNN-based feature extractions usually require a square input resolution [84]. Accordingly, uniformly scaling the original image to a standard size is needed before feeding them to the prediction network [20]. We have created a collection of basic datasets by resizing all images into rectangles with a height and width of 416 and 416, respectively. Thus, all selected D-CNN-based methods were trained in the 416×416 pixels versions but not in the original resolution. Note that there is an exception to the smallest images required by EfficientDet that was restricted to 512 × 512 pixels.

### 3.6. Evaluation Methodologies

Based on past studies of DL-based barcode recognition, several common performance metrics were used to ensure the accuracy and performance of DL methods. In this study, the detection accuracy of all D-CNN methods was investigated using Mean Average Precision (mAP). In addition, the runtime is used to evaluate and confirm the influence speed of the models. The definition and principle of the key evaluation metrics are given as follows:

Mean average precision (mAP) is often used as a standard metric to evaluate the accuracy and robustness of DL methods in object detection tasks. It can be calculated according to the Average Precision (AP) of different classes and then averaged over a number of classes [85]. As shown in Equation (2), AP is obtained by measuring pairs of precision (P) and recall (R) values for different ranks [32].

(1) $$\mathrm{AP}=\sum_{n}\left(R_{n}-R_{n-1}\right)P_{n},$$

(2) $$\mathrm{mAP}\;=\frac{1}{N}\sum_{n}^{N}{\mathrm{AP}}_{n},$$

In this aspect, P is the fraction of barcodes correctly recognized by the D-CNN models over the actual number of all barcodes that the model can recognize. However, R represents the probability of accurately detecting ground truth barcode images. Hence, mAP can be further calculated by Equation (2), resulting in the possible value from 0 to 1. The highest mAP score, the most accurate the model is in its detection.

For the comprehensive study, the IoU has also been explored for all experimental scenarios. IoU is a quantitative measure to quantify how the ground truth and predicted boxes match. It can be defined as the ratio of Area of Overlap (represents the interaction of the true ground box and the bounding box of the regression result) to the Area of Union (represents the union of the truth box and the bounding box of the regression result) [86]. Specifically, IoU is used as a threshold to classify whether the prediction is true positive or false positive [21]. The performance of the D-CNN methods in this study was investigated and compared across different IoU thresholds. This technique avoids the ambiguity of choosing the optimal IoU threshold for evaluating the accuracy of the competitive models. The definition of IoU is denoted in (3).

(3) $$\mathrm{IoU}=\frac{\mathrm{Area}\;\mathrm{of}\;\mathrm{Overlap}}{\mathrm{Area}\;\mathrm{of}\;\mathrm{Union}},$$

The IoU is equal to 0 means 0% overlap between the predicted and the ground truth box. Whenever the IoU is 1, there is an exact match between the two boxes. Thus, the higher the IoU, the better the prediction.

## 4. Results and Discussion

### 4.1. Dataset Statistics

**Figure 3:**
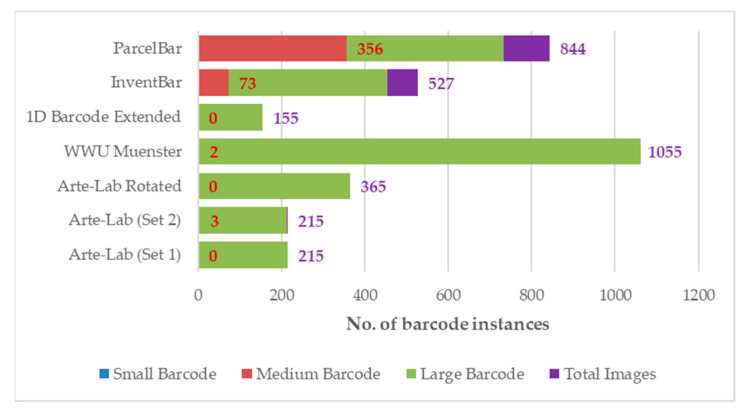
Number of annotated barcode instances classified by barcode size: small, medium, and large.

Following that, we analyze the key properties of the InventBar and ParcelBar datasets compared to all benchmarking barcode datasets. Figure 3 reveals the fraction of annotated barcode instances in each dataset. We observed that each of the benchmarking datasets varies significantly in size (number of images contained in the dataset) and differed in the number of barcode instances, falling within the small, medium, or large categories. There are no existing small-sized barcodes for all datasets, while the medium-sized barcodes appeared very few (only two to three instances) in WWU Muenster and Arte-Lab (Set2), respectively. Simply said that the number of images in the series of Arte-Lab datasets, 1D Barcode Extended, and WWU Muenster is at the same level as their annotated barcodes. This means almost all images in the datasets, as mentioned earlier, contained only a single barcode. In contrast, our new datasets include the captured images with either one barcode tag or multiple barcode tags, which leads the D-CNNs to enhance their detection capabilities for similar objects located in the same image. We emphasized that the multiple barcode instances per image will be useful for training complex D-CNN methods to detect barcodes more precisely.

It is common knowledge that all object detection algorithms would perform well on large objects, especially in the event that the models were previously trained on larger objects [87]. Smaller objects are typically harder to localize and require higher contextual reasoning to recognize. Similar to our case, all the adopted D-CNNs were pretrained using an MS COCO dataset encompassing 640 × 480 pixels images [80], while the training and testing on the real barcode data have been done over 416 × 416 pixels images. As seen in Figure 3, the InventBar and ParcelBar datasets contain loads of barcode instances classified as medium-sized, while all barcodes from other datasets are considered large barcodes. Therefore, it is unsurprising that all D-CNN methods applied over both datasets show comparatively lower detection accuracy because the models prefer larger barcodes. In this aspect, we can conclude that our proposed datasets contribute some distinguishing characteristics that could not be observed in other existing datasets. InventBar and ParcelBar were created by addressing one of the critical challenges of object detection algorithms with various sizes of barcode objects over the real-world foreground and background images.

### 4.2. Barcode Recognition Accuracy

In order to verify the quality of barcode datasets, this paper compares five different D-CNN algorithms over seven competitive datasets with an image resolution of 416 × 416 pixels. In-depth analysis of the barcode recognition accuracy, the mAP was evaluated by considering the overlapping percentage between the ground truth barcode region and the prediction boundary boxes of barcode. In this regard, recognition accuracy would reflect the degree to which the D-CNN methods can correctly detect or localize one or more barcode instances that appeared in the image. The higher the accuracy rate, the better performance of the detection solution. At the same time, we use IoU threshold values to indicate different levels of detection confidence. First, we quantify the mAP at the IoU threshold = 0.5, denoted as mAP@0.5 (i.e., there is only a 50% overlap between the two regions). Straightforwardly, if the prediction boundary captured over 50% overlap with the ground truth barcode region, the prediction was considered a successful match. For the more challenging detection task, secondly, we set the detection confidence of all comparative models ranging from 0.5 to 0.95, indicated mAP@(0.5–0.95) (i.e., considering 50%–90% overlap between the predicted and the actual barcode region) by increasing every 0.05 and reporting an averaged result.

In Table 6, we collected and summarized the best recognition accuracy of different D-CNN methods. The D-CNNs were applied over the two proposed datasets and several other popular datasets, including Arte-Lab Medium Barcode (Set 1), Arte-Lab Medium Barcode (Set 2), Arte-Lab Rotated Barcode Dataset, 1D Barcode Extended Dataset, and WWU Muenster. Compared to other D-CNN methods and with mAP@(0.5–0.95), YOLO v5 presents a higher mAP for all benchmarked datasets. These results show that the YOLO v5 can detect barcode objects more accurately. It can also imply that YOLO v5 is the most robust model in the SCM domain since it provides a good result even measured with a high degree of matching confidence. The tendency of mAP measured in all datasets is obviously in the same direction. Leastwise, the results obtained from the two invented datasets do not deviate from the comparative ones.

Conversely, when a 50 percent overlap between the predicted and the actual barcode is considered, the mAP of both YOLO v5 and YOLO x displayed the lowest value for almost all datasets except InventBar. The reason is that YOLOs perform a greater number of detection errors than the existing D-CNN methods. In addition, the YOLOs network often struggles to detect small and adjacent objects from each grid with only two bounding box regions [88]. Interestingly, when the D-CNN models were applied over the two proposed datasets (InventBar and ParcelBar), none of the models reached 1.0 mAP. On the other hand, D-CNNs applied on the remaining datasets do have. The characteristics of the benchmarking datasets apparently biased the model training to detect barcodes, particularly at IoU 0.5 easily. This means that the model acknowledges the perfect match at only half of a barcode tag is detected. Either at IoU 0.5 or IoU 0.5–0.95, however, the mAP results tested on InventBar and ParcelBar are more reasonable. This evidence proves that our datasets are scene-based and exhibit unique characteristics that brought all adopted models to fall into a higher challenge than the other datasets.

**Figure 4:**
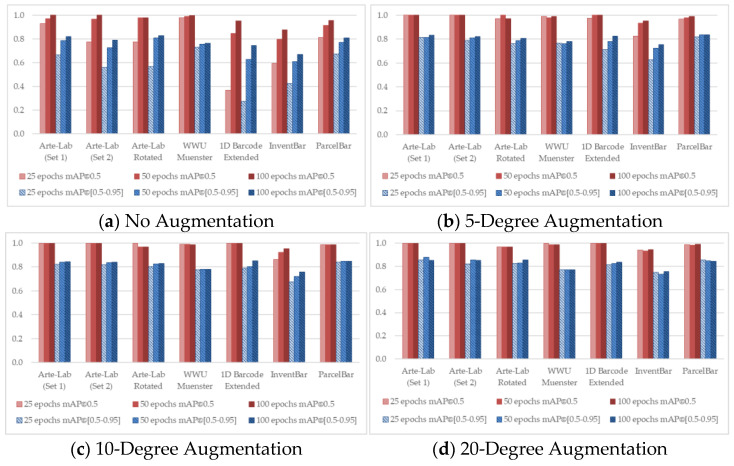
Recognition rate of EfficientDet applied over seven public barcode datasets.

**Figure 5:**
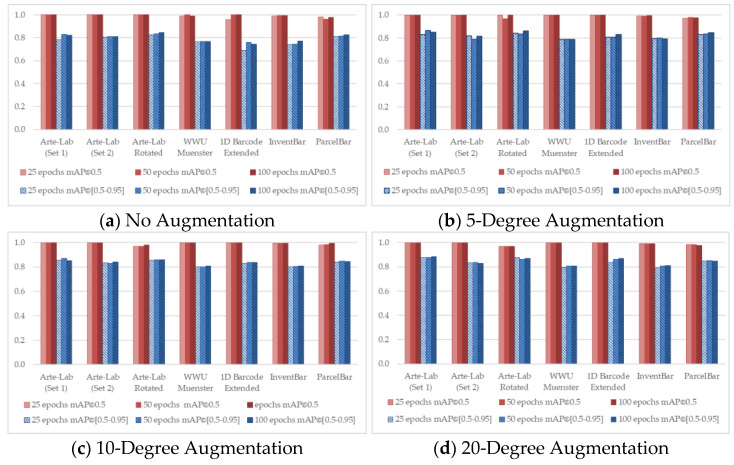
Recognition rate of Faster R-CNN applied over seven public barcode datasets.

**Figure 6:**
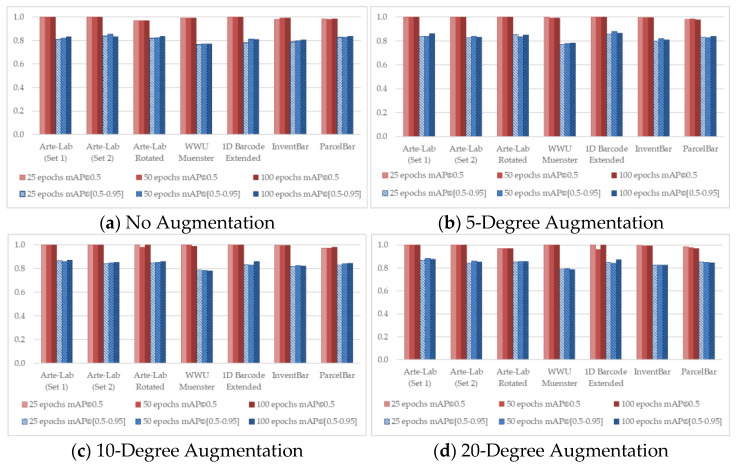
Recognition rate of RetinaNet applied over seven public barcode datasets.

**Figure 7:**
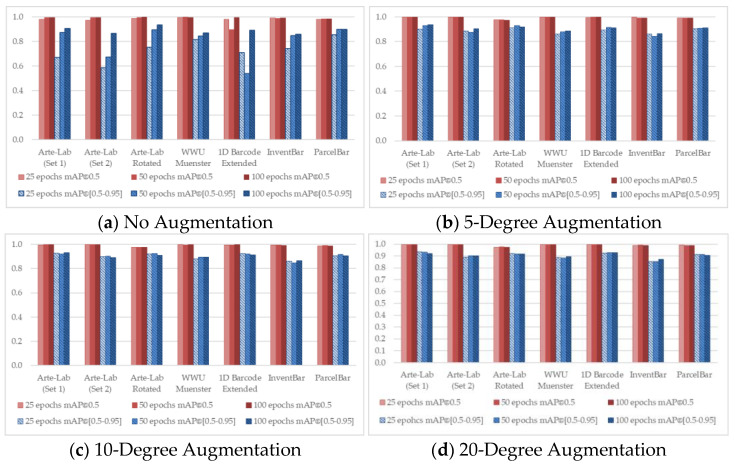
Recognition rate of YOLO v5 applied over seven public barcode datasets.

**Figure 8:**
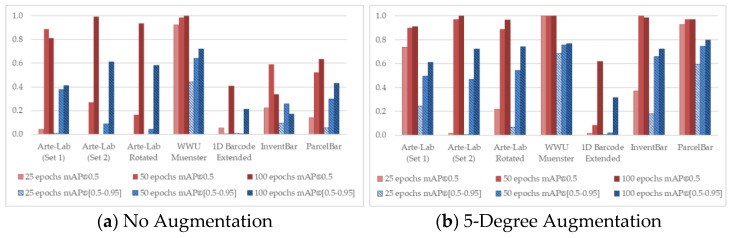

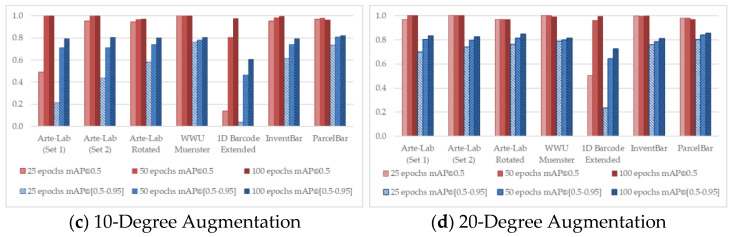
Recognition rate of YOLO x applied over seven public barcode datasets.

To observe the detailed characteristics of different D-CNN methods over seven benchmarked datasets, we conducted the training process by taking advantage of different experimental configurations. Figure 4, Figure 5, Figure 6, Figure 7 and Figure 8 demonstrated the barcode recognition rate (mAP@0.5 and mAP@(0.5–0.95)) of EfficientDet, RetinaNet, Faster R-CNN, YOLO v5, and YOLO x, respectively. In corresponding to what has been described in Section 3.5, we also quantified and reported the mAP results based on the augmentation degree. For each set of illustrations, the mAP values from the two IoU thresholds were calculated at different epoch intervals, i.e., 25, 50, and 100, shown as follows.

Considering all experimental scenarios illustrated in Figure 4, Figure 5, Figure 6, Figure 7 and Figure 8, the best mAP@0.5 achieved the perfect barcode recognition capability during the training. However, the average mAP@(0.5–0.95) is always lesser since the models rely on a higher overlapping percentage between ground truths and the precited ones. Although the mAP results from different D-CNN methods are varied, the overall results gradually improve with the increased degree of augmentation settings (~10 to 20). This evidence confirms that the augmentation approach dramatically boosts the overall D-CNN performance and decreases overfitting. When more augmentation degree is considered, the execution results of the models are slightly better at a higher number of epochs (~50 to 100), as can be observed in Figure 4, Figure 5, Figure 6, Figure 7 and Figure 8 (d). On different image-augmented distributions, the detection accuracy observed in InventBar and ParcelBar is nearly stabilized. Their mAP variations were very small when tested on a large number of epochs with intensively augmenting the images, except only in the case of YOLO x, which shows massive fluctuations.

When focusing on the models, RetinaNet and Faster R-CNN are less sensitive to the weight parameters, i.e., epochs number, augmentation degree, and IoU. Another important observation is that the results of both RetinaNet and Faster R-CNN are almost similar in all experimental scenarios. This situation highlights the performance and stability of some underexplored methods such as RetinaNet when applied to a new application domain. Apart from the YOLO x, utilizing all employed methods practically benefits detecting barcodes and is also possible for our two invented datasets.

From the experiments, we were able to perceive that detecting barcodes in the SCM domain should be done with a high degree of detecting confidence, and YOLO v5 is the best solution among all employed methods. It is proved that some of the D-CNN methods that were previously used in different domains, e.g., YOLO v5, EfficientDet, and RetinaNet, can be precisely applied in a new SCM environment. Apart from the performance of D-CNN approaches, the unique and real-world characteristics of recent public barcode datasets in the field are also key influences challenging the barcode recognition tasks. However, the originally embedded features of real-world barcode images are sometimes insufficient for the learning process. Increasing the epoch numbers and augmentations is a way to enhance the model training process and improve the model’s accuracy in detecting barcode images. This is a vital issue that needs to be considered since better barcode localization results consequently lead superior positive impact for decoding barcode information in the actual SCM industry, e.g., reducing operation mistakes/decoding errors, increasing speed, and saving cost. Hence, this investigation recommends that researchers or practitioners should train and test the D-CNN-based barcode recognition methods with sufficient learning iterations and loops of transformations.

### 4.3. Runtime Performance

In this section, we evaluate the effect of D-CNN methods on each dataset based on runtime performance at the optimal accuracy results (mAP@(0.5–0.95)). To verify the tendency of time required to complete the training process, we also present the performance of each model from the dimensions of average runtime.

As illustrated in Table 7, YOLO v5 has shown the greatest runtime performance in a series of ArteLab Barcode Datasets, while EfficientDet can recognize barcodes and learn faster than other methods for WWU Muenster, InventBar, and ParcelBar. This evidence reflects the outstanding performance of these two D-CNN models in providing high detection accuracy but using comparatively low effort. In the dimension of average runtime shown in Table 8, YOLO x outperforms other D-CNN methods in all datasets. This result causes no doubt for us because YOLO x is the latest object detection solution adopted in this study. It is well-known for reducing computational costs and improving inference speed. One can also see that all D-CNN methods spent much more time training the WWU Muenster, InventBar, and ParcelBar, most of which required up to an hour to complete the training task. These large datasets are ranked as the top three with the highest barcode images. Thus, we assumed that the more extensive the barcode dataset, the more time is required to train the models. One more interesting point is that the size of ParcelBar is slightly larger than WWU Muenster (both contain a very close number of barcode images). However, the time consumed for D-CNN methods on ParcelBar is always lesser than the time spent training the WWU Muenster. Clearly, the dataset size is not only a key influence on time complexity but also includes the image properties, e.g., a certain amount of barcode tags, image background, and illumination. These characteristics would have a large effect on the model’s performance.

**Figure 9:**
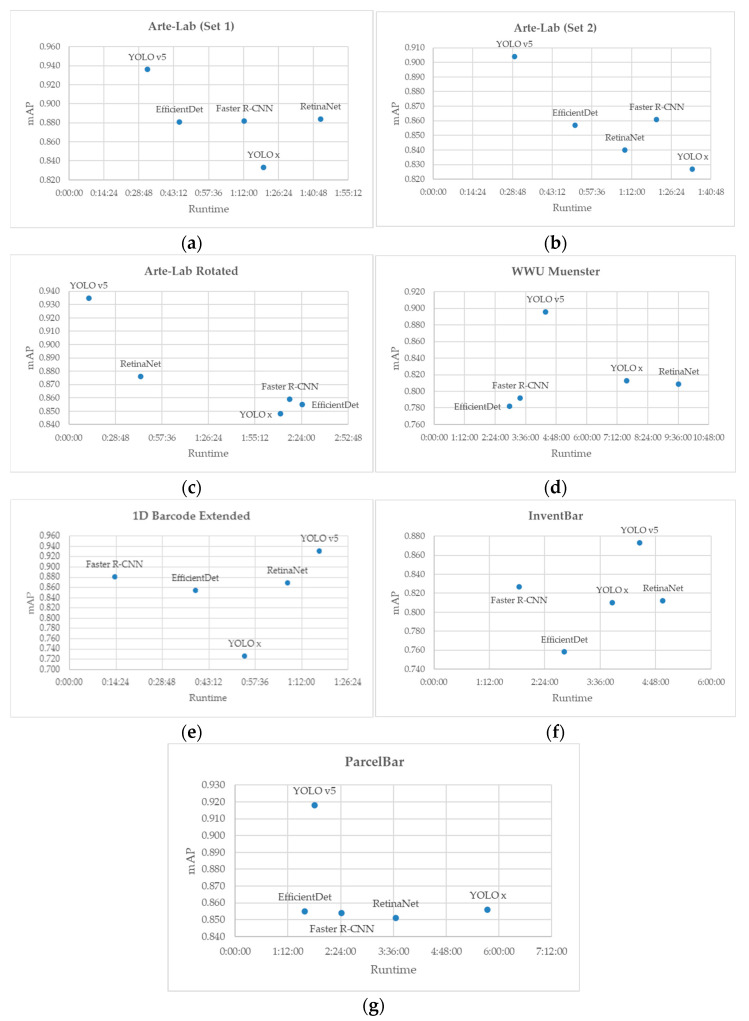
Runtime performances of the D-CNN methods applied over seven public barcode datasets: (**a**) Arte-Lab Medium Barcode (Set 1), (**b**) Arte-Lab Medium Barcode (Set 2), (**c**) Arte-Lab Rotated Barcode, (**d**) WWU Muenster, (**e**) 1D Barcode Extended, (**f**) InventBar, and (**g**) ParcelBar.

At this stage, we also explore the correlation between the accuracy result defined by mAP and the runtime performance of different D-CNNs on each dataset. From Figure 9, all D-CNN methods satisfy high detection accuracy with reasonable runtime. We can clearly see that one of the YOLO v5 is always positioned at the left-hand side of the scatter chart, excluding the 1D Barcode Extended dataset and InventBar. Compared to the competitive methods, the position of YOLO v5 implies a high accuracy with a negligible drop in runtime. It is noticeable that YOLO v5 consistently outperforms YOLO x in either accuracy or execution time or both, as shown in Figure 9a–g. Our experimental result is consistent with the study from Gillani et al. 2022 [89], who confirmed the higher AP of YOLOv5 than that of YOLO x. We emphasize that using YOLO v5 on ParcelBar, WWU Muenster, and a series of Arte-Lab datasets will greatly benefit the model training in both accuracy and time dimensions. For our proposed InventBar, although YOLO v5 has the highest accuracy, it requires a higher time consumption. Regarding this issue, Faster R-CNN is highly suggested to apply on the InventBar with the hope of increasing opportunity for real-time barcode detection in the SCM.

### 4.4. Application Effects of D-CNNs on 1D Barcode Recognition

For the sake of completeness, we continually discussed the application effects of different D-CNN methods on the 1D barcode recognition, as summarized in Table 9. Through mainstream single-stage D-CNN network models, EfficientDet and RetinaNet have never been explored in the barcode detection domain. EfficientDet is a scalable object detection method, as it can be applied to a wide range of resource constraints. Its network architecture can be optimized by jointly scaling up network width, depth, and resolution. The model seems better at detecting 1D barcodes in a large dataset, i.e., WWU Muenster and ParcelBar, with an excellent running speed but comparatively low accuracy. Under similar accuracy constraints, EfficientDet most often outperforms RetinaNet only at the cost of inference speed. This is because the RetinaNet considers hard samples (e.g., extreme foreground-background images) plus two task-specific subnetworks that yield high detection accuracy as close to the two-stage detectors’ performance but still taking a long runtime.

One can be observed that the best detection accuracy achieved by EfficientDet, RetinaNet, and the two-stage Faster R-CNN sticks together at the same level. This situation reflects the two-stage detectors, i.e., Faster R-CNN is not always practically benefitting the barcode detection in the SCM domain, even though many previous studies in barcode recognition have proven it. Faster R-CNN uses region proposals to localize barcode objects within the images instead of looking at the complete image, thus providing fairly good barcode detection accuracy and runtime.

Among all comparable D-CNNs, YOLO v5 shows the most distinguishing characteristics. The method falls within a single convolutional network model to predict the bounding boxes and the class probabilities for the boxes. It is a hyperparameter evolution method containing multiple variants, thus having size and inference time tradeoffs. Notably, YOLO v5 can improve the training convergence time for 1D barcode detection while increasing model accuracy. The model seems suitable for detecting barcodes from small to large volumes with a broad range of barcode sizes and image qualities.

In contrast, YOLO x performs less accurately but much more speed (average runtime) than others in almost all datasets even though it is the latest improved method adopted in this study and was claimed for a new high performance exceeding previous versions of the YOLO family [24,90]. YOLO x uses decoupled head architecture instead of coupled head to separately perform the classification and localization processes by aiming at higher accuracy achievement. Nevertheless, the experimental results show that the method needs higher computational efforts to achieve the best detection accuracy. This might be due to the size of the YOLO x model being larger than YOLO v5, and the model contains a greater number of parameters (9 million parameters for YOLO xs and 7.2 million parameters for YOLO-v5s [90]). Another assumption is that the YOLO x model was introduced using strongly augmented data helping the model to generalize and rely on more features. However, some data augmentations from YOLO x might not be appropriate for detecting real-world barcode images. Intuitively, overstepping augmented barcodes and limiting epoch number at 100 maximum from our experiments might be key reasons affecting the model to decrease the accuracy. Therefore, using still images and increasing training iterations appear to be the more useful setting for the YOLO x.

From the detailed analysis above, it is undeniable that the performance of D-CNNs depends on both network architecture and training settings. All methods can detect barcodes with high accuracy but largely differ in learning speed. In the SCM environment, 1D barcode detection must be further improved to meet zero detection error, especially in real-time detection. Therefore, designing a more effective D-CNN model considering various key influential factors such as image features, dataset characteristics, and barcode recognition environment would be a great challenge to barcode recognition development.

## 5. Conclusions

This work proposed the problem of D-CNN-based barcode recognition for supply chain management. In this context, reliable and fully completed barcode datasets are required to model and enhance the recognition capability of the D-CNN solutions. This work put forward the two innovative barcode datasets: InventBar and InventBar, by considering barcode images attached to consumer goods and parcel boxes in the express delivery warehouse. The proposed barcode data were from real-life images collected from an indoor warehouse and without simulated data presented in the datasets. Five state-of-the-art and underexplored D-CNN models were trained and tested over the two proposed datasets, together with other publicly available barcode datasets. The performance of each model was analyzed both in terms of mAP and runtime. Benchmarking experiments on all datasets showed that YOLO v5 performs comparatively better than other methods, especially when the optimal accuracy rate is on our focus. The mAP@(0.5–0.95) of YOLO v5 achieved an average of 0.913 from all datasets and reached the maximum of 0.936 in ArteLab (Set 1). Comparing runtime performance at the best mAP results, EfficientDet spent less time recognizing barcode instances in large datasets, i.e., in WWU Muenster, InventBar, and ParcelBar. Instead, YOLO x has shown to be the fastest model when the average runtime of all experimental scenarios is considered. When investigating the relationship between runtime and accuracy, YOLO v5 works best on our ParcelBar (mAP@(0.5–0.95) = 0.918) while reasonably satisfying the barcode detection with relatively low training time requirements (nearly one time faster than the other four D-CNN models). Hence, we can confirm the feasibility of using YOLO v5 with the ParcelBar dataset for detecting barcodes with sufficient speed and accuracy. For InventBar, however, the Faster R-CNN is highly suggested, especially when the time dimension is put as a first priority. To this end, our study also contributes to the notion that some D-CNN methods, mostly adopted in different but unrelated domains, could precisely expand to the realm of possibility in the SCM application. For future work, real-time barcode localization and decoding in a smart warehouse or SCM environment should be investigated to make the D-CNNs more effective for barcode recognition. On this basis, two possible implementation solutions should be considered. First, implementing either a novel or an improved D-CNN-based barcode recognition on still images and head-up images (the well-prepared barcode image datasets) should be enhanced to flawless accuracy at real-time detection. Second, the application of small barcode or far away barcode detection from remote sensing technologies and aerial objects, i.e., drones in the indoor warehouse, should also be explored in the next research. Under the conditions of satisfying the performance requirements of the D-CNNs on 1D barcode recognition, future research could be designed by taking into account the scanning technologies, aerial image features, type of barcodes, and warehouse environment.

## Figures and Tables

**Table 1:** Current publicly available barcode datasets.

Name	Size	Resolution (Pixel)	Instance per Image		Image Feature		Barcode Annotation	
			Single	Multiple	Synthetic	Real-Life	Provided	No. of Annotations
Arte-Lab Medium Barcode (Set 1)	215	640 × 480	✓			✓		
Arte-Lab Medium Barcode (Set 2)	215	640 × 480	✓			✓		
Arte-Lab Rotated Barcode	365	640 × 480	✓	✓		✓		
1D Barcode Extended	155	648 × 488	✓	✓		✓	✓	155
WWU Muenster	1055	640 × 480	✓	✓		✓		
Dubská M.	400	604 × 402	✓		✓		✓	400
Sörös G.	320	720 × 1280	✓			✓	✓	328
Bodnár-Synthetic	10,000	512 × 512	✓		✓			
Bodnár-Huawei	98	1600 × 1200	✓		✓	✓		
Percentage			100%	33.33%	33.33%	77.78%	33.33%	

**Table 2:** D-CNN-based barcode recognition methods employed over 2015–2021.

Authors	Year	D-CNN	Public Dataset	Private Dataset	Accuracy
Chou et al. [61]	2015	CNN	CypherLab		0.952
Grzeszick et al. [34]	2016	CNN		Product on the racks	0.470
Li et al. [62]	2017	Faster R-CNN	ArteLab WWU Muenster		0.989 0.994
Hansen et al. [33]	2017	YOLO v2	ArteLab Rotated WWU Muenster Dubska’ M. Sörös G.		0.914 (all)
Zhang et al. [63]	2018	SSD		Medical Label	0.945
Tian et al. [64]	2018	R-CNN	ArteLab WWU Muenster	Mixed Barcode	0.963 (ArteLab and Muenster) 0.925
Ventsov and Podkolzina [65]	2018	CNN		Ventsov N.N	0.974
Zhao et al. [66]	2018	CNN		Barcode-30k	0.942
Ren and Liu [67]	2019	SSD	ArteLab WWU Muenster CipherLab		0.885 0.884 0.992
Yang et al. [68]	2019	CNN		Fashion Label	0.967
Xiao and Ming [69]	2019	YOLO v2	ArteLabWWU Muenster		0.912 0.939
Pu et al. [11]	2019	CNN		Production line	0.991
Zhang et al. [70]	2019	Fast R-CNN	ArteLabWWU Muenster Dubska´ M. Sörös G.		0.871 (all)
Blanger and Hirata [71]	2019	SSD		Blanger L.	0.770
Yuan et al. [72]	2019	R-CNN	CipherLab COCO Val2017	UAV123 Yuan, B.	0.999 (all)
Li et al. [73]	2019	DSC		DPM Code QR Code Images	0.999 (all)
Suh et al. [35]	2019	YOLO v2	ArteLab Rotated WWU Muenster	15 Carriers Shipping Labels	0.980 (all)
Kalinov et al. [32]	2020	CNN		UAV barcode	0.961
Brylka et al. [14]	2020	YOLO v3	ArteLab ArteLab Roated WWU Muenster		0.870 (both ArteLabs) 0.860
Jia et al. [51]	2020	Faster R-CNN	ArteLab WWU Muenster Dubska´ M. Sörös G. Bodnár-Synthetic	Jia, J.	0.834 (all)
Zhang et al. [74]	2020	Fast R-CNN	ArteLab WWU Muenster Dubska´ M. Sörös G	Zhang, J.	0.879 (all)
Tan [36]	2020	CNN		Logistic Robot Barcode	0.988
Zharkov et al. [75]	2020	CNN		ZVZ-Synth ZVZ-Real	0.967 (all)
Suh et al. [37]	2021	CNN		Shipping Labels	0.997
Do and Pham [38]	2021	YOLO v3	COCO Val2017	Supermarket Products	0.900 (all)
Zhang et al. [15]	2021	YOLO v4		Liwei Z.	0.906

Remark: Convolutional Neural Network (CNN), Region-based Convolutional Neural Network (R-CNN), Single-Shot Detector (SSD), Depth-wise Separable Convolution (DSC), and You-Only-Look-Once (YOLO).

**Table 3:** Number of different-sized barcode regions contained in InventBar and ParcelBar.

Dataset	No. of Images	No. Barcode Regions in Different Sizes			No. of Annotations
		Small	Medium	Large	
InventBar	527	0	73	454	527
ParcelBar	844	0	356	732	1088

**Table 4:** Pretrained backbone network architectures used for D-CNN methods.

Authors	D-CNN Methods	Backbone
Tan et al., 2020 [25]	EfficientDet	tf_lite0
Ren et al., 2016 [27]	Faster R-CNN	resnet50_fpn_1x
Lin et al., 2018 [26]	RetinaNet	resnet50_fpn_1x
ultralytics/yolov5, 2022 [23]	YOLO v5	small
Ge et al., 2021 [24]	YOLO x	yolox_s_8x8

**Table 5:** General information of the benchmarked datasets and sub-datasets.

No.	Dataset	Training Set	Validation Set	Test Set	Total
1	Arte-Lab Medium Barcode (Set 1)	86	86	43	215
2	Arte-Lab Medium Barcode (Set 2)	86	86	43	215
3	Arte-Lab Rotated Barcode	146	146	73	365
4	WWU Muenster	422	422	211	1055
5	1D Barcode Extended	62	62	31	155
6	InventBar	337	338	169	844
7	ParcelBar	210	211	106	527

**Table 6:** The best barcode detection accuracy of different D-CNN methods applied over all benchmarked datasets.

D-CNN-Based Methods	Arte-Lab (Set 2)		Arte-Lab (Set 1)		Arte-Lab Rotated		WWU Muenster		1D Barcode Extended		InventBar		ParcelBar	
	mAP 0.5	mAP 0.5–0.95	mAP 0.5	mAP 0.5–0.95	mAP 0.5	mAP 0.5–0.9	mAP 0.5	mAP 0.5–0.9	mAP 0.5	mAP 0.5–0.9	mAP 0.5	mAP 0.5–0.9	mAP 0.5	mAP 0.5–0.9
EfficientDet	1.000	0.881	1.000	0.857	1.000	0.855	0.999	0.782	1.000	0.854	0.954	0.758	0.991	0.855
Faster R-CNN	1.000	0.882	1.000	0.861	1.000	0.859	1.000	0.792	1.000	0.880	0.997	0.827	0.985	0.854
RetinaNet	1.000	0.884	1.000	0.840	1.000	0.876	1.000	0.809	1.000	0.869	0.994	0.812	0.994	0.851
YOLO v5	0.998	0.936	0.998	0.904	0.996	0.935	0.998	0.896	0.998	0.930	0.996	0.873	0.994	0.918
YOLO x	1.000	0.833	1.000	0.827	0.970	0.848	1.000	0.813	0.996	0.726	0.998	0.810	0.981	0.856

**Table 7:** Runtime performances of D-CNN methods at the optimal detection accuracy.

Datasets	EfficientDet	Faster R-CNN	RetinaNet	YOLO v5	YOLO x
Arte-Lab (Set 1)	0:45:28	1:12:20	1:43:49	**0:32:17**	1:20:11
Arte-Lab (Set 2)	0:51:24	1:21:05	1:09:27	**0:29:30**	1:34:02
Arte-Lab Rotated	2:24:35	2:16:44	0:44:22	**0:12:20**	2:11:01
WWU Muenster	**2:57:38**	3:23:15	9:36:58	4:22:45	7:34:19
1D Barcode Extended	0:39:08	**0:14:06**	1:07:39	1:17:35	0:54:28
InventBar	**1:10:40**	1:50:38	4:57:20	4:27:19	3:51:31
ParcelBar	**1:35:02**	2:25:25	3:38:58	1:48:22	5:44:34
**Total runtime ^1^**	**10:23:55**	**12:43:33**	**22:58:33**	**13:10:08**	**23:10:06**

^1^ The runtime performance at the optimal detection accuracy is acquired by mAP@(0.5–0.95) and is presented in hh:mm:ss.

**Table 8:** Average runtime performances of D-CNN methods applied over seven public barcode datasets.

Datasets	EfficientDet	Faster R-CNN	RetinaNet	YOLO v5	YOLO x
Arte-Lab (Set 1)	0:24:25	0:35:05	0:26:20	0:26:41	**0:20:40**
Arte-Lab (Set 2)	0:25:54	0:40:14	0:31:13	0:27:32	**0:22:52**
Arte-Lab Rotated	0:36:39	0:56:25	0:41:57	0:41:27	**0:31:53**
WWU Muenster	2:05:51	2:21:52	2:17:29	1:57:52	**1:49:48**
1D Barcode Extended	0:15:36	0:22:51	0:17:25	0:19:23	**0:13:52**
InventBar	1:03:21	1:32:39	1:10:38	0:59:40	**0:53:27**
ParcelBar	1:30:38	2:19:18	1:43:05	1:39:07	**1:19:54**
**Total runtime ^2^**	**6:22:24**	**8:48:24**	**7:08:07**	**6:31:42**	**5:32:26**

^2^ The average runtime performance was calculated from all experimental scenarios and represented in hh:mm:ss.

**Table 9:** Application effects of D-CNN methods on 1D barcode recognition.

D-CNN Methods	Model Type	Effects on 1D Barcode Recognition
EfficientDet	Sigle-stage	The model seems better at detecting 1D barcodes in large datasets, i.e., WWU Muenster and ParcelBar, with a small running speed. At the same detection accuracy level, EfficientDet is often faster than RetinaNet. The method required less time than other methods at the best accuracy result. It saved at least two hours during the inference process on all barcode datasets. Thus, the method might be practically applied for detecting a large number of barcode instances in various warehouses when time is limited.
Faster R-CNN	Two-stage	The model’s overall detection accuracy and running speed are moderate compared with other D-CNN solutions. Faster R-CNN tends to perform relatively fast on large datasets containing a number of medium-sized barcodes, i.e., InventBar, ParcelBar, and WWU Muenster.
RetinaNet	Single-stage	RetinaNet yields high detection accuracy as close to the performance of Faster R-CNN. Considering the optimal accuracy constraint, RetinaNet consumed lots of time as similar to YOLO x. RetinaNet might contribute to complex background images or real-time barcode detection rather than still and simple barcode images.
YOLO v5	Single-stage	YOLO v5 can decrease training time while increasing barcode detection accuracy. The model might be suitable for detecting 1D barcodes, either a small or large dataset. YOLO v5 is considered robust even if applied on a board range of barcode sizes or far away barcode objects and image qualities.
YOLO x	Single-stage	YOLO x performs less accurately but much more speed than other D-CNNs. The method needs higher computational efforts, i.e., time and iteration numbers, to achieve the best detection accuracy. Still or real-world captured images without or less augmentation might be one of the more useful settings for the YOLO x.

## Data Availability

Publicly available datasets were analyzed in this study. This data can be found here: https://cmu.to/BenchmarkBarcodeDatasets (accessed on 13 October 2022).
